# The fear of social stigma experienced by men: a barrier to male involvement in antenatal care in Misungwi District, rural Tanzania

**DOI:** 10.1186/s12884-022-04383-x

**Published:** 2022-01-17

**Authors:** Maendeleo Boniphace, Dismas Matovelo, Rose Laisser, Victoria Yohani, Hadija Swai, Leonard Subi, Zabroni Masatu, Sylvia Tinka, Hannah Faye G. Mercader, Jennifer L. Brenner, Jennifer L. Mitchell

**Affiliations:** 1 Catholic University of Health and Allied Sciences (CUHAS), P. O. Box 1364, Mwanza, Tanzania; 2 Bugando Medical Centre (BMC), P. O. Box 1464, Mwanza, Tanzania; 3 Ministry of Health, Community Development, Gender, Elderly and Children (MoHCDGEC), Mwanza, Tanzania; 4 Cumming School of Medicine, University of Calgary in Canada, 3280 Hospital Drive NW, Calgary, Alberta T2N 4Z6 Canada

**Keywords:** Fear, Social stigma, Maternal health, Rural Tanzania

## Abstract

**Background:**

Evidence has shown that male involvement is associated with improved maternal health outcomes. In rural Tanzania, men are the main decision makers and may determine women’s access to health services and ultimately their health outcomes. Despite efforts geared towards enhancing male participation in maternal health care, their involvement in antenatal care (ANC) remains low. One barrier that impacts men’s participation is the fear and experience of social stigma. This study, builds on previous findings about men’s perspectives in attending antenatal care appointments in Misungwi district in Tanzania, examining more closely the fear of social stigma amongst men attending ANC together with their partners.

**Methods:**

Twelve individual interviews and five focus group discussions were conducted using semi-structured questionnaires with fathers and expectant fathers. In-depth interviews were conducted with health providers, volunteer community health workers and village leaders. Interviews were audiotaped, and transcripts were transcribed and translated to English. Transcripts were organized in NVivo V.12 then analyzed using thematic approach.

**Results:**

Three main themes were found to create fear of social stigma for men: 1. Fear of HIV testing; 2. Traditional Gender Norms and 3. Insecurity about family social and economic status.

**Conclusion:**

Respondent’s experiences reveal that fear of social stigma is a major barrier to attend ANC services with their partners. Attention must be given to the complex sociocultural norms and social context that underly this issue at the community level. Strategies to address fear of social stigma require an understanding of the real reasons some men do not attend ANC and require community engagement of community health workers (CHWs), government officials and other stakeholders who understand the local context.

## Introduction

Maternal mortality is a critical health issue in Low- and Middle-income countries (LMICs) such as Tanzania. In Tanzania, maternal mortality ratio has remained high, at about 566 per 100,000 live births [1], despite several interventions at the health facility and community levels. Over the decades, research has shown that addressing gender equity issues in LMICs is an integral part of the efforts to reduce maternal and perinatal mortality and morbidities [2, 3]. Men are often decision makers at family level in terms of when and how women access and utilize antenatal care (ANC) services and ultimately maternal and child health outcomes [4, 5].

Enhanced male participation in ANC services, can increase shared decision making in impactful health choices such as parenting, health care-seeking for delivery and illness, contraception, and family planning. Increased male involvement during pregnancy has several advantages such as in reducing maternal stress through emotional, logistical, and financial support. Furthermore, male participation during ANC is associated with increased use of health facility delivery and use of postnatal health services and reduced postpartum complications [3, 6]. Although men play a key role as the main decision makers in the family, interventions and programs to improve access and utilization of maternal services, often target women, and despite efforts to include and encourage men’s attendance, their involvement in maternal health matters remains low [5].

Studies in Nigeria and Gambia, patriarchal countries similar to Tanzania, revealed that social stigma is one factor that contributes to men’s low attendance to maternal services. In many communities, community members look down on men who escort their wives to ANC services [5, 7, 8]. In a study in Kenya, men reported that they would be perceived as being “ruled” by their wives, “ridiculed” and not seen as “men” if seen taking part in maternal services [9]. Further, economics and material insecurity have been found in other studies to be a barrier to male attendance. A study in Geita region of Tanzania found that community members may feel stigmatized for not having good attire at the health facility and is a barrier for ANC seeking [10].

The social stigma related with HIV infection and HIV testing which is mandatory to both partners when attending ANC services can prevent male attendance for maternal services [11, 12]. While men fear being tested, they also fear that their results will not be kept confidential; healthcare worker’s (HCWs) who broke confidentiality has been found in South Africa [13]. Such experiences can create mistrust, fear and resistance for men to attend maternal services [8, 10, 14, 15].

Men are clearly impacted by various cultural practices and gender roles, and these may negatively impact their involvement in maternal health. In our recent study [2] we examined perceptions of males in Misungwi district that impact attendance to ANC services and described two themes: 1. Perceived exclusion during ANC visits among men and 2) Traditional gender norms resulting to low attendance among men, with subthemes of (a) shame and (b) secrecy. In this study, we sought to more closely examine and better understand the social stigma, which appeared in our subtheme of shame, and how the fear of social stigma may impact male ANC attendance.

## Methods

This study was a secondary analysis of qualitative data collected for a broader research study of perceptions of male involvement in maternal services in Misungwi District located in Tanzania [2]. This qualitative study aimed to fully examine stories of shame, stigma or fear, which affects men’s ANC attendance together with their partners. This study was nested as a sub-study within a larger longitudinal implementation and evaluation of the Mama na Mtoto project intervention in rural Tanzania [16] which aimed to improve the delivery of essential health services to pregnant women, mothers, newborn and children under-five in Misungwi and Kwimba districts.

### Study setting

Misungwi District is among seven districts of Mwana Region. It consists of 2579km2 with population of 351,607 according to the 2012 national census (NBS, 2012). The district is located in the Northwestern part of Tanzania, 45km from Mwanza town. The district has a predominantly rural population (91%) and a majority of population are Sukuma tribe, (91.9%,) speaking their tribal language in addition to Swahili (Tanzania National language). The district is divided into four divisions two semi-urban and two rural. The major economic activities are cattle-keeping and subsistence farming. Misungwi district has 2 hospitals, 4 health centres and 45 dispensaries. Two communities (“divisions”) were selected for the study, due to their high maternal mortality [17].

### Study design

This qualitative study was informed by the ecological framework and the framework influenced sampling, data collection and analysis. Ecological frameworks consider the individual, interpersonal, community and societal factors and recognize the complex interplay across all levels of a health problem and the influence on health behaviors. Using an ecological framework sensitized our examination of barriers to male ANC attendance to multiple factors.

### Sampling procedure

Interview and focus group participants were recruited using purposive sampling method. From the four divisions of Misungwi district we purposively choose two rural divisions (Mbarika and Inonelwa) based on its unfavorable MNCH indicators [17]. In the divisions we conveniently selected one ward in Mbarika and three wards in Inonelwa. At the ward level, sampling procedures were culturally sensitive and tried to foster safety and trust in the communities. As such the following steps were taken:

- Initial contact was made with the village executive officer (government leader) in the village/community to explain the purpose of the study.
- The village government leader arranged orientation meetings with village officials and community health workers (CHW)
- Next a public meeting was held to inform community members about the study aim and selection criteria of participants. The meeting was intended to build trust, outline participant criteria, and ensure voluntary participation.
- Criteria for participation included being a male over the age of 18, who had a partner experiencing their first pregnancy, males with one or more children, no cognitive disability, and a permanent resident living in Misungwi district for over 6 months. Key informants selected included village leaders (possible influencers of health service uptake), volunteer CHWs, and health providers.
- Meetings were held with participants who had volunteered to participate. Consent was discussed and documented, including confidentiality of the data and the right to withdraw at any time. Dates and location of interviews were discussed with participants.

### Data collection

The research team developed an interview guide in advance incorporating personal experiences of team members, relevant literature, and questions aimed at target different levels of the ecological model. This tool was piloted in a different rural environment with men of similar characteristics, and small modifications and probes were added to the guide. Questions included “how does your community perceive men who attend ANC appointments with their partners?” and what were your experiences or what have you heard about attending an ANC visits?” Sukuma speaking research assistants were recruited to assist in obtaining consent and data collection for non-Swahili speaking participants. In total, five focus group discussions and 12 in-depth interviews were conducted with a total of 50 participants.

#### Focus group discussions (FGD)

The five FGD were composed only of fathers and homogenous by age to promote comfort and build on common emergent themes. The focus groups lasted on average of 60-90 min and took place at quiet and convenient places agreed by participants in their locations/homes. Each FGD consisted of 8-12 participants. There were 15 men whose partners were pregnant for the first time and 29 fathers who had one or more children. Their age ranged from 25 to 60 years old.

#### In-depth interviews (IDI)

Individual interviews were conducted with one health care provider, three village leaders and two community health workers (CHWs). The individual interviews lasted on average of 40-60 min each and took place at participants homes or at a secured room in the village office as per participants choices. Additionally, fathers from the FGD were selected for individual interviews to provide more in-depth feedback to meet saturation. Six men took part making a total of 12 individual interviews conducted.

In both FGD and IDI, the facilitators conducted semi-structured interviews with participants, interviews were recorded, and research assistants’ rote field notes and documented non-verbal cues that provided a secondary source of data. Interviews conducted in Swahili were transcribed and then translated in English while those in Sukuma were transcribed in Swahili and later translated in English. All approached participants who agreed to join the study participated.

#### Quality checks

Quality checks for the transcripts were performed by research team members who were not involved in data collection through listening to audio and reading corresponding transcript and noting any errors. Data were later reviewed by researchers who had conducted the interviews, they re-read all transcripts while listening to the audio recordings for the purpose of further validating the transcripts for accuracy and language.

### Data analysis

Despite that data analysis began during interviews and team meetings after the interviews, the secondary thematic analysis was influenced by our analysis and findings of our first paper 10.1186/s12884-021-03585-z [2] and we used sensitizing concepts such as fear, social stigma and gender norms to more fully explore the experience of social stigma. We used NVivo version 12 to organize qualitative data. Transcripts were read line by line and chunks of data were assigned a code. The codes were organized in NVivo, and code books were generated, reviewed and agreed upon by research team. Codes were organized and collapsed into broader themes. Memos were made to describe the rationale and process of sorting codes into the themes. Research analysis discussed discrepancies about themes until consensus was reached. The main themes were later obtained upon agreement with the research team.

## Results

The table below shows demographic characteristics of respondents whose age ranged from 25-60 years and the majority were above 35 years (70%) and married (80%) as described in the Table 1 below.

**Table 1:** Demographics of study participants (*N* = 50)

Characteristics	N	%
**Age (years)**		
25-34	15	30
≥35 years	35	70
**Marital status**		
Married	40	80
Common law relationship	10	20
**Education**		
Primary	41	82
Secondary	9	18
**Occupation**		
Peasant	42	84
Community Health Workers	2	4
Healthcare Workers	2	4
Village Leaders	3	6
Driver	1	2

The three themes found in this study include: 1. Fear of HIV testing, 2. Gendered male norms and roles and 3. Insecurity about family social and economic status.

### Theme 1: Fear of HIV testing

In a standard ANC practice, men and women are required to take a HIV test when attending ANC services. Respondents revealed that men fear taking the HIV test and the social stigma that accompanies having a test, testing positive and compromised confidentiality about test results. The fear of HIV is a collective fear, accompanied by social stigma in the community; however, respondents share that women may be disproportionately bearing the responsibility of being tested. Respondents shared that men may prefer their partners to attend ANC, take the HIV test and as a result many men will assume that they are HIV negative if their partner has HIV negative result.

One respondent explains, “*Men are unwilling to be tested for HIV. They just tell their women, you go alone, if you are HIV negative, I am also negative”* (Father with one child- IDI). Given that HIV testing is mandatory during ANC visits, male involvement in this portion of maternal care is subsequently impacted more than other aspects of maternal and newborn care.

The fear of HIV testing is augmented by the fear that others will overhear their diagnosis or that healthcare workers breach confidentiality and may reveal a positive HIV test in front of others or share it with others without consent. Several respondents shared experiences that increase their fear of being stigmatized. One participant noted that confidentiality was not honored in the clinic:

*“Some community members noticed that I was seated somewhere privately with a healthcare worker. To my surprise they asked the nurse about the conversation and this nurse revealed to him about my result. Next time I will not tell her a thing”* (Father with more than one child-FGD).

*“Help us with the health services we receive from our healthcare providers, health providers should not reveal the secrets of the patients because when they do that, that person won’t tell them a thing when they meet her/him again, even if she has some illnesses, she/he won’t tell them because they are not trustworthy”* (Father with one child-FGD).

Another respondent shared that community members may know the results of a man’s HIV test, and they assert that healthcare workers are sharing this information and breaching confidentiality. Some individuals noted that the physical space is often not conducive to private conversations, increasing the risk of men experiencing discomfort in taking the HIV test and desire better spaces at health facilities. One respondent explained:

*“The government should create more privacy so that there can be space to accommodate people to talk freely with a health provider so that what you tell her remains there and you do not hear about it elsewhere.”* (Expectant father-IDI).

### Theme 2. Gendered male norms and roles

Participants shared several impacts of locally held beliefs about male and female roles in the community that can create a fear of social stigma for men. Attending and being part of ANC is often considered a ‘woman’s issue’ and it not considered to be a man’s role to be involved in ANC services. Many men report feeling shame and a fear of being laughed by their fellow men if they escort their partners to the health facility. A father shared, “w*e feel ashamed, shame is upon us we men to attend ANC clinic. The shame comes when we escort our wives, other men will laugh at you…”* (Father with more than one child-FGD).

Those identifying as Sukuma ethnicity, described some beliefs that men are being controlled or charmed by their partners, which conflicts with their roles as a man, if they attend ANC visits with their partner and some men did not want to be perceived, by other men, as being controlled: A man explained:

*“The community believes that if you’re with your wife all the time people will say (heeee) this man has been whipped, set under control of a woman that’s why we are not going*” (Father of one child-FGD).

When couples arrive at the clinic, there may be further discomforts for men and women. Culturally, it is not common for men and women to sit together in most gatherings. The father report that.

*“Sitting together in the waiting spaces or on the same bench is “uncomfortable to sit with many pregnant women…while there are no other men.”* (Expectant father-FGD).

Lastly, having multiple sexual partners among men is seen as an accepted norm in study communities. Yet, there was a perception amongst men that while having a concubine is common, it is commonly still done in secrecy. As a result, men avoid attending ANC appointments with women to avoid being seen by others with someone who is not their wife.

*You find most of men have more than one family, so if he escorts a concubine and that man is seen by other people at the clinic, it will be revealed that he escorted another woman, that will become a barrier to escort a woman to ANC. Because men fear family conflict which may arise if he is seen with a concubine at the health facility* (Father of one child-IDI).

*“You find someone went out of his marriage then the woman faces challenge to come with him to ANC clinic as others will know, that’s why they pick other men such as “boda bodas drivers” to satisfy health providers that they came with their husband”* (Health care provider-IDI).

### Theme 3. Insecurity about family social and economic status

Respondents reported experiences that contributed to insecurity, shame, and fear of being judged by health facility workers or community members. Some respondents disclosed that for Sukuma speakers, not being able to understand or speak Swahili contributed to their insecurity and resistance to attend the health facility. One participant described:

*“In my opinion some men do not escort their partners because they are afraid of being asked questions at the health facility, so they are shy and unconfident of expressing themselves in Swahili language if the HCW does not communicate tribal language (Sukuma)”* (Father with more than one child- FGD).

In the Sukuma community, people commonly dress in their best clothes to attend health care facilities; also, families are expected to bring clothes (Khanga) and other supplies for birthing. In Sukuma culture, it is important for men to be the provider and are considered the ‘head of the household’ and being perceived as unable to provide for their wife and baby is a large barrier in attendance to ANC services. Men whose families lacked economic means to provide desirable clothing and birth supplies were reported to avoid facility visits because of lacking material resources and associated feelings of inadequacy. A village leader shared that there is insecurity around not having the means to arrive in nice clothes:

*“Some men do not escort their pregnant woman because first of all they do not take good care of them in terms of buying nice clothes, so they feel ashamed when they go together with a woman who has no proper attires”* (Village Leader-IDI).

## Discussion

We found several experiences about the fear of social stigma that serve as a barrier to men’s attendance and involvement in ANC in rural Tanzania. There is a social stigma surrounding HIV testing and testing positive for HIV, which is augmented by fears of a lack of confidentiality around test results. Engrained gender norms impact men’s attendance, such as gendered roles (e.g., pregnancy is a woman’s issue; sitting with women at the clinic), social stigma related to appearing “charmed” by one’s partner or shame about being judged as being of a lower economic status (e.g., being unable to provide clothes/materials for their partner). While extramarital affairs are culturally common among men, they likewise are done in secret and men fear being seen by others should they escort a woman who is not their wife to the health facility. To maximize male attendance, efforts must work with communities to understand and address deep-rooted cultural and gender-based norms that create fears for men and seek local solutions to encourage meaningful participation during pregnancy in locally appropriate ways.

While men attending maternal services with their pregnant partners is increasingly being endorsed as a standard to promote optimal family-centered care, its achievement in many communities in rural Sub-Saharan Africa, has been hard to accomplish [2, 6]. While our study presents fears of social stigma which may be unique to the Sukuma tribe, our themes are consistent with other countries which have identified similar barriers to maternal services [10, 18]. Similar to other traditional gender normed communities, pregnancy is perceived as a woman’s issue [14, 19] and the social stigma against men who partake in “women’s work,” such as ANC visits, or who appear controlled by their partner [19] hinders male engagement in maternal health in Misungwi district. Moreover, in Sukuma ethnic culture, it is not common for men and women to sit together at gathering, and as such there is discomfort in sharing physical spaces and benches with women at the health facilities. Similar experiences men’s discomfort were found in Uganda [8], and in South Africa, men were sometimes told by health care workers to go outside because the space is only for women [20].

Our study echoes other studies where locally held beliefs about gender result in embedded and rigid masculine norms [12, 21, 22]. In Sukuma communities, men are the providers for the family; our study found that fear of social stigma among community members in Misungwi was attributed to the lack of appropriate clothing during ANC visits and being perceived as a poor provider. Issues of poverty impacting the uptake of ANC services have likewise been found in Geita, Northern Tanzania [10]. While having multiple sex partners in several parts of Tanzania is perceived as normal practice by men, for women it is perceived as prostitution [23] and can create conflict within the family if discovered. As such men keep their extramarital affairs secret and refrain from attending appointments with women with whom they are having affairs; similar to the study in Uganda, they fear being seen, found out, judged by HCWs and want to avoid conflict with their wife or family members [24].

The social stigma associated with HIV and HIV testing at health facilities, has been identified as a barrier for male attendance to maternal services [8, 10, 12]. Men in our study fear being tested, but also fear that their results will be disclosed without their consent. Lack of confidentiality and trust among HCW’s has, likewise been found in South Africa [13]. The social stigma of HIV and HIV testing combined with the lack of confidentiality experienced by community members has strong implication on the uptake of maternal health services. It impairs dignified respectful maternity care to the clients [8, 10]. This may in turn form mistrust for HCWs competencies, as a result the community members may decide not to seek health care services to their facility and may lead to malpractices which have legal implications to HCWs at the health facility [14, 15].

This study involved perspectives of a variety albeit relatively small sample of men and health workers from a narrow demographic group (rural communities; over 25 years old). Our sample may have missed men who may have characteristics and logistics that make it difficult for them to attend ANC services, such as those working full time, working away from the community, or men anxious about their personal situations and disclosure (i.e., polygamy, age difference, casual partner relationship). The strength of our study is that it has considered rural population where male ANC attendance with partners is low [25, 26]. Qualitative data collection allowed us to explore men’s perspectives on a topic which is culturally not considered to be their role and to explore the sensitive topic of their fears of social stigma.

## Recommendations and Conclusion

This study contributes to the growing body of work looking at factors that deter men from attending ANC visits, therefore it is important for policymakers, government, development partners, and maternal program implementers who strive to integrate men into visits to enhance family-centered care. In rural settings in Tanzania, increased male attendance at ANC will not increase solely because of policy or encouragement. Interplay of several factors is required for the change in male engagement in maternal issues to occur apart from clearer understandings of the local source of stigma that might inhibit men’s ANC attendance, and strategies that are respectful of cultural and gender norms. This study suggests incorporating locally relevant strategies to encourage men’s attendance, such as local government meetings that incorporate an agenda to discourage unfavorable gender norms through community-based education and role modeling at various levels of local government and community. Community health care workers (CHWs) and “male champions” can help to educate men and model for men, the importance of attending ANC services with their partners, while also specifically addressing and validating their fear of social stigma. The health facility management can consider health facility environment which supports men’s needs during ANC and delivery, such as adherence to confidentiality, sensitivity and strategies that address language barriers and programs that provide supplies to those who cannot afford them.

Addressing uptake of maternal and newborn services in culturally bound communities requires understanding of the social stigma apart from several factors experienced by men accompanying their partners during ANC visits to achieve an overarching goal, as stipulated in this study, to help in formulating interventions that are tailored to community needs in rural settings in Tanzania. While social stigma may vary from one community to another, men must be heard and understood within their local context, with community solutions developed to acknowledge and challenge sources of stigma.

## Data Availability

For data and materials used in this study can be requested from the project principal investigator through this contact: Dr. Dismas Matovelo at magonza77@yahoo.co.uk.

## Abbreviations

ANC: Antenatal Care
BMC: Bugando Medical Centre
CHW: Community Health Workers
CUHAS: Catholic University of Health and Allied Sciences
FGD: Focus Group Discussion
HCW: Health care workers
HIV: Human Immunodeficiency Virus
IDI: In-depth Interviews
KII: Key Informant Interviews
MoHCDGEC: Ministry of Health Community Development Gender Elderly and Children
UoC: University of Calgary
